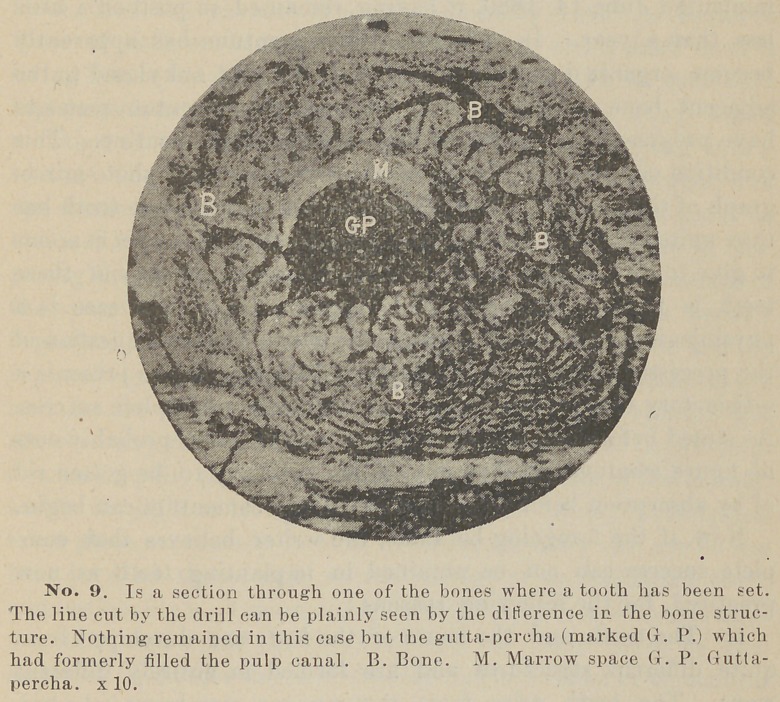# Some Notes on Experimental Implantation of Teeth

**Published:** 1891-01

**Authors:** M. H. Fletcher

**Affiliations:** Cincinnati, Ohio


### THE DENTAL REGISTER.

VOL. XLV.] JANUARY, 1891. [No. 1.

### COMMUNICATIONS.

### Some Notes on Experimental Implantation of Teeth.

BY M. H. FLETCHER, D.D.S., M.D., CINCINNATI, OHIO,

Read before the Ohio State Dental Society, October 8th, 1890.

This paper is a report of an incomplete series of experiments on the implantation of teeth. Though the experiments are incomplete they were carried far enough to indicate at least one important fact, namely, that the pericementum probably plays no part in the repair and retention of implanted teeth, but that such success as is attained is due to the re-organization of the cementum.

In order that the writer's position may not be misunderstood, it seems necessary to define a few terms which have been employed in explaining the success and failure of implanted teeth in the present status of the operation. Terms will be used in this paper with the following meanings :

CASES OF FAILURE.—To be understood as including those in which implanted teeth have been lost from any cause within six weeks from time of insertion.

PARTIAL SUCCESS.—Including those cases in which the teeth have not become firm after a few months, and which show continual signs of local inflammation.

SUCCESS.—Including those cases where the implanted tooth has become apparently healthy and is usable, and in most cases perfectly rigid.

COMPLETE SUCCESS.—Including those cases which may be found to be perfectly healthy and usable after a period of seven to ten years.

The history of implantation need not be repeated since it is familiar to all. But to speak of the various ideas and methods that have been followed is directly in point, for these show that we have been relying upon the wrong tissue for the succes of the operation.

For instance, Dr. W. J. Younger, the originator of the process, believes that in order to be successful the peridental membrane should be intact on every part of the root. He has used both fresh and old teeth, but I believe uses old nearly altogether. He reports a large per cent. of successful cases.

Dr. George Cunningham is reported as saying in 1888: "Thus far I have used nothing but freshly extracted teeth, and believe that if a tooth is implanted before the death of the pericementum and cementum the prospect of union is increased, and the loss by absorption decreased. The general opinion seems to have prevailed that freshly extracted teeth would be more successful in implantation than old ones."

It seems to have been an almost universal opinion that the pericementum must be intact in order that these cases might be at all successful, the writer of this paper having held that opinion.

On the other hand, Dr. G. L. Curtis, who reports a very large number of successful cases, strips the entire membrane from the root, believing it to be a hindrance rather than a help in their repair. However, he gives no physiological reason for his opinion. So far as my knowledge goes Dr. Curtis is the only person on record who takes this view, and operates by denuding the roots of the teeth. And it would seem, according to the results of the experiments herein reported, to be a step in the right direction.

Dr. H. A. Smith's paper, entitled "Dental Implantation," read before the American Dental Association in August, 1889, and the discussion of the same, probably gives the latest and most reliable statistics and opinions on this subject. Computation from this paper shows a loss of about 17 per cent. of the cases reported. This is the nearest that can be calculated from the returns given. But it is probably far from correct, since so few who are known to have performed the operation responded to Dr. Smith. The probabilities are that there are many more cases of failure than have been reported. Also there probably is a large number who have performed the operation who have never been heard from in any way. This is more likely to be true especially if the results of their experiments were unsatisfactory. Dr. Smith's notes are compiled from operations performed prior to August, 1889.

**No. 1.** The illustrations accompanying this article are partly of the normal tissue for the purpose of comparison with those involved in the cases of implantation. Figure 1 represents normal dentine and cementum and the Junction between them. D. T. Dentinal Tubes. I. S. Interzonal Layer. C. S. Cement Cells. x 175.

The writer's own experience on the human subject is limited to six cases. One of these was a failure, three were partially successful, and two successful. But we consider even the successful cases as far from complete success, since they to-day show signs of inflammation in the region of the roots, in addition to a tendency of the gums and bone to recede from the neck of the tooth. They can be made to bleed easily by a moderate pressure of the finger on the gum over the tooth. These few cases are not sufficient to establish a rule, but the consideration of their symptoms in connection with the physiological processes probably involved in their repair tends to establish this opinion without further evidence.

The mystery connected with the subject of repair in implantation, and the various opinions expressed upon it, induced the writer to begin a series of experiments on the lower animals with a view of determining, if possible, what the real conditions are. These experiments you will readily understand were fraught

No. 2. Represents a section of normal bone, the cells of which are C. to be comp. and with the cement cells in figures 1 and 6. H. Haversian canals. x 350.

with great difficulties in the absence of the knowledge of any preceding experiments in the same line.

After a conference with other histologists on the subject we decided to use a sheep for the first experiment, and the incisor teeth of small dogs for material. These teeth were selected because of their small size, thereby reducing the extent of the cutting necessary to perform the operation.

In the beginning the plan of procedure was to insert one or two teeth at a time, at periods of one to two months apart, thereby expecting to have for examination a complete series of the various stages of repair from its incipiency to its completion.

The experiments were begun April 16, 1889, by extracting the incisor teeth of a small cur. These were dried before the fire for several hours, after which the pulp chambers were thoroughly cleansed and disinfected, then filled with chlora-percha and gutta-percha nerve canal points.

**No. 3.** Normal Structure *in situ* from the goats jaw, to be compared with Nos. 4 and 5. C. Cementum. P. C. Pericementum. H. C. Haversian Canals. B. Bone. x 50.

April 19. Armed with the teeth and instruments for the experiment we repaired to the field of labor. When ready to begin, the greatest difficulty presenting itself related to the means by which the teeth could be held in position long enough for them to become fastened so that they might not drop out. Several plans had been considered. But when ready to proceed they were all abandoned, and a new one, suggested by my assistant, Dr. Arthur Knight, was adopted. This consisted of sewing the skin entirely over the teeth, the implantations all being made externally instead of in the mouth. I had intended to operate in the mouth, but abandoned the attempt on account of the extreme difficulties in the execution.

The first attempts were made on a sheep about $2\frac{1}{2}$ years old. The animal was put under the influence of sulphuric ether, and the skin was laid open over the external surface of the cannon bone of the right hind leg one inch below the upper joint. A

**No. 4.** Section through an implanted tooth showing surrounding tissue and junction with bone at J. B. Bone. C. Cementum. D. Dentine. P. C. Pulp Canal, filled with gutta-percha. M. Marrow space. x 10.

hole was drilled with a large fissure burr, endeavoring if possible to extend the opening into the cancellous portion of the bone, and yet in such a way as not to injure the joint through producing inflammation therein. One half of the crown was then cut off from a tooth in order that its point might not irritate the skin. After it was settled in the drilled socket we took two stitches in the incision in the skin drawing it together so as to cover the tooth completely, thus preventing it from dropping out when it became loosened by inflammation. After having dusted iodoform plentifully over the wound and into the hair of the vicinity the operation was considered completed.

A 1 to 1000 solution of bichloride of mercury was used freely on hands, instruments, and teeth, as well as in the wounds, during the operations.

A second tooth was inserted in the opposite hind leg in the corresponding position in the corresponding bone. The same

**No. 5.** Represents a section through same tissues. x 50 showing their structure more plainly. D. Dentine. C. Cementum, (to be compared with the cement cells in Fig. 1,) P. C. Pulp Canal filled with gutta-percha, B. Bone, where the ridged union with the tooth occurs.

plan of disinfecting, removing a part of the crown, and sewing up the skin, was followed in the second operation, as it was indeed in all subsequent implantations.

The animal revived slowly from the anæsthetic. It did not recover but died on the third day from what appeared to have been congestion of the lungs.

Thinking that a goat, being a more hardy animal, would be better adapted for the experiment, one was secured which was about three years old.

April 29. The goat was secured to a bench, and given something to eat instead of being anæsthetized. One tooth was inserted in each hind leg in substantially the same manner as was done in the case of the sheep. Within a few days considerable inflammatory exudation occurred in the region of the wounds but this terminated by resolution in its normal time.

**No. 6.** Represents a section taken from the place of union between implanted tooth, with bone. B. Bone. D. Dentine. C. Cement cells. x 340.

No further attempt was made at implanting until June 14, when we inserted a tooth in the lower border of the inferior maxillary bone, left side, about two inches anterior to the angle. Another attempt was made at this time to insert a tooth inside the mouth, into the lower maxillary, but it was entirely unsuccessful. The remaining teeth weie all inserted in the lower border of the inferior maxillary bone on either side, and about $\frac{1}{2}$ inch apart, in the same manner as before described. The cuts in the skin healed in the normal time for such tissue to repair.

The operations were performed on the following dates:

1889, April 26, 2 teeth.
" June 14, 1 tooth.
" July 27, 1 tooth.
" August 8, 2 teeth.
" November 8, 1 tooth.
1890, January, 17, 2 teeth, making in all nine teeth implanted.

**No. 7.** Section of normal structure through one of the dog teeth, such as were used to implant, showing only a single row of cement cells. D. T. Dentinal Tubes. C. Cement Cells. B. Bone. x 600.

Only two of the operations in the jaw produced any swelling. In one of these a point of the drill was broken off and remained in the bone, and in the other the tooth was allowed to play very loosely in the socket as a matter of experiment. This tooth was one which had been denuded of pericementum, the only one so prepared. The escape of these foreign bodies from the bone to the skin probably produced the swelling in these cases.

On May 3, 1890, one year and two weeks from the date of the first operation on the goat, the animal was killed, and the bones containing the implanted teeth were dissected out and examined.

On removing the skin from the bones it was found that some of the teeth remained with the crown above the bone. But they were invariably covered with periosteum, and all but two apparently had undergone complete decalcification as far as could be

**No. 8.** Represents a section through the tooth in which the hypertrophied bone had inclosed the enamel above the neck of the tooth and the absorption on two sides. B. Surrounding bone. M. Marrow spaces. D. Dentine. E. Enamel P. C. Pulp Canal filled with gutta-percha. x 10.

judged from outside appearance. The periosteum covering the crown of the tooth and that, projecting into the socket, as well as that in the immediate neighborhood, seemed free from inflammation. The bone immediately adjacent to each tooth showed signs of having been inflamed and had become hypertrophied to an extent that left the tooth or its remnants in a funnel-shaped depression.

Some of the teeth seemed to have disappeared completely; nothing remaining to be seen of two of them but the gutta-percha which had been used to fill the root canal. This was found underneath the pericementum just over the site of implantation.

Blocks of bone containing the teeth or their remains were now sawed from the body of the bone and placed, some in alcohol, some in Muller's fluid, where they remained for two weeks, and

**No. 9.** Is a section through one of the bones where a tooth has been set. The line cut by the drill can be plainly seen by the difference in the bone structure. Nothing remained in this case but the gutta-percha (marked G. P.) which had formerly filled the pulp canal. B. Bone. M. Marrow space G. P. Gutta-percha. x 10.

were then cut into hard sections by filing and polishing, and are here represented in photo-micrographs. Two are being decalcified, for sections with the microtome.

On making sections some seem to have been completely decalcified, yet with very little or no characteristics of either dentine, cementum, or enamel remaining. In three some dentine was left. One of them had enamel upon it which had become surrounded by periosteum, it having grown into the socket. But the periosteum was not attached to the tooth. Absorption of the root had progressed until fully half of it had disappeared, the absorption having gone up two sides of the tooth into the crown. A photo-micrograph from this specimen, as well as other sections, will be shown so that the matter may be better understood.

The most interesting section made is one cut from the tooth implanted June 14, 1889, it having remained in position a little less than a year. In this case the cementum has apparently become organized on two sides and thoroughly ankylosed to the adjacent bone. And not only this but the cementum seems to have progressed in growth at the expense of the dentine. This condition can be seen plainly in the accompanying photo-mirco-graph of the section. Now while the fact that this one tooth has thus united does not prove that all others will do so, yet it seems to give to us a hint as to how the retention and rigidity of these teeth is to be explained. This termination of this case is a physiological process and explains the much discussed question of the processes which occur in implanted teeth. It also presents a satisfactory explanation of their permanency or complete success. As stated before in this paper the pericementum probably cuts no figure whatever in their repair but is simply to be gotten rid of by absorption before organization of the cementum can begin.

Now, if the foregoing be facts, the writer believes that complete success can not be obtained in implanting teeth as now practiced, for the following reasons:

1. The teeth and the bones of the body are developed from quite different structures and are formed in entirely different ways. The teeth arise from the mucous membrane (the epiblast), while the bones originate from the periosteum, the marrow, and hyaline cartilage (the mesoblast). Dentine is formed by a stationary cell, the odontoblast, of which only the processes become surrounded by dentine while the bone is formed by the osteoblast, a floating or migrating cell, which itself finally becomes incorporated in the bone tissue. But you may say the cementum is not dentine. And neither is it bone. It has few or no Haversian canals, and derives its nourishment from the pericementum, the office of which we believe is to produce and nourish cementum. Our experiments seem to show, however, that cementum can be increased without the membrane. If it were a fact that the dried pericementum on the roots of implanted teeth took a new life and performed its functions as it once did, then would we have a complete success of these operations.

If it were possible to implant immediately freshly extracted teeth, then the chances of success probably would be equal to those of skin or bone grafting. Or if we could take from a living foetus the dental follicle and implant it with its surrounding tissue into another and older jaw, we probably would stand a chance of its retaining life and ultimately developing into a tooth. The latter case, of course, is totally impracticable, and the former almost as much so, for, if the proper teeth could be obtained of suitable size and shape the necessary delay in preparing the root canal would most likely be fatal to the success of the operation. Again, supposing the cementum does become organized, as we attempt to show, it can but grow and be replaced by new material at the expense of the dentine of the root in the same manner that older Haversian systems are resorbed and replaced by new ones. This process of bone resorption and repair, probably continues during the life of the animal, and in the light of biology it can only be so. Now, if resorption takes place continually in vital tissure it certainly can not be less true in dead tissue, such as the dentine of an implanted tooth unquestionably is. Consequently it would only be a few years until the entire root would be absorbed and its place taken by new material. Whether this new material would be bone or cementum we do not know, but it most likely would ultimately be bone, for it would be built by bone-producing tissues, the cementum having in time become absorbed. The fate of transplanting and replanting is not yet forgotten. And yet these operations came nearer the necessary requirements than does implanting.

While preparing the present notes my attention has been called to a report of some experiments bearing directly upon the points in question, that of the organization of dead bone, which we believe to be comparable to the organization of dead cementum. They were carried on by William B. Hopkins, M.D., and C. B. Penrose, M.D., Ph.D., of Philadelphia, and published April 5, 1890, in the *Journal of the American Medical Association.* These experiments were to determine the practicability of the use of sterilized dead bone dowels in the union of fractures. These dowels were sterilized by first boiling them thoroughly and then keeping them in an alcoholic solution of corrosive sublimate (1 to 1000) ready for use. Their methods of procedure were to drill through or into the bones of young dogs and insert a sterilized bone peg which exactly fitted the hole. This was done both in normal and fractured bones. To give the results I quote from their article as follows:

Experiment No. 1.

"The extremities of the dowel which were in contact with the shaft of the femur had become thoroughly organized, being full of Haversian canals continuous with those of the dog bone of which they had become a part, and on fresh sections showed the pink line of living vascular bone.

The intervening portion betwen these extremities, corresponding to the medullary cavity, showing no attempt at organization, as it was pure white on fresh section, and contained no Haversian canals, but the erosions on its surface clearly indicated that rapid absorption was taking place. x x x

From these experiments they give the following deductions:

1. That where sterilized dead bone is placed, under favorable circumstances, in contact with living bone it undergoes organization.

When, on the other hand, it is acted upon by periosteum, it is absorbed, and when placed in the medullary cavity in not too large bulk, organization combined with absorption takes place.

2. That these processes go on, perhaps, most actively between the fifth and eighth weeks, and are not necessarily associated with any inflammatory action.

3. That therefore, where these dowels are employed to pin together fragments of bones after fracture, to fix the extremities of bones after resections, or for any other mechanical purpose in surgery to which they are adapted, they may be relied upon to do their work for a period of one month or six weeks, and hence to give ample time, as a rule, for union to occur.

After this their presence being no longer required they gradually lose their identity in the surrounding bone and disappear."

As to absorption and organization our experience was identical with the experiences of Drs. Hopkins and Penrose in that the tendency to absorption was much greater when the tooth extended into the marrow cavity, and a tendency to organization where compact bone structure surrounded the teeth, and that absorption proceeded more rapidly in places where the periosteum came into contact with the tooth than when this membrane was absent—in other words, when the teeth came into close proximity to tissues which produce or foster the growth of giant cells or osteoblasts the absorption was greatest, and that organization is most likely to occur when the tooth is fitted only moderately tight into solid bone structure.

Another point bearing on the practicability of the subject is brought out by experiments carried on by Mr. Wm. Scovell Savory in 1864. He demonstrated by a series of experiments performed upon various animals that ivory pegs driven tightly into healthy bone were more quickly absorbed than if the fit were only moderately tight. Our own experiments up to the present would not have brought this point out. But the hint is pertinent and I think should be heeded by those performing the operation in the future.

In the light of our past experiments we can see the following points which tended to make them less successful:

1. The teeth used were of young animals and had a very thin growth of cementum on the roots.

2. The teeth were fitted too tightly into the bone.

3. They were completely covered with the skin instead of leaving the crowns protrude.

In the continuation of the work these points can all be noted so as to obtain a greater number of desired results.

From the foregoing we have the following conclusions:

First. From the statistics we conclude that the operation probably has fallen into disrepute either from failure or lack of confidence.

SECOND. That when teeth are implanted the roots of which are covered with dried periosteum, this membrane must be absorbed before union can take place, and that when union does occur, it is most probably that of ankylosis between vascular cementum and the surrounding bone. In view of this fact, if the operation is to be performed, such teeth should be selected as have a considerable layer of cementum, and should be denuded of the old membrane before being implanted.

THIRD. That organization of the cementum is most favored when it is so placed in living bone that it impinges—but not tightly—in all its parts upon solid bone, and for this reason the cicatrix of bone formed by that growth which fills the socket after extraction of a tooth, is the most favorable place for the implanting of teeth.

FOURTH. That the resorption and rebuilding of the tissues of the body necessitate the absorption of the dentine of the roots of implanted teeth, and thereby their loss. But that as a temporary replacement of lost teeth the operation of implantation is justifiable to those who comprehend it to be such.

## Figures and Tables

**No. 1. f1:**
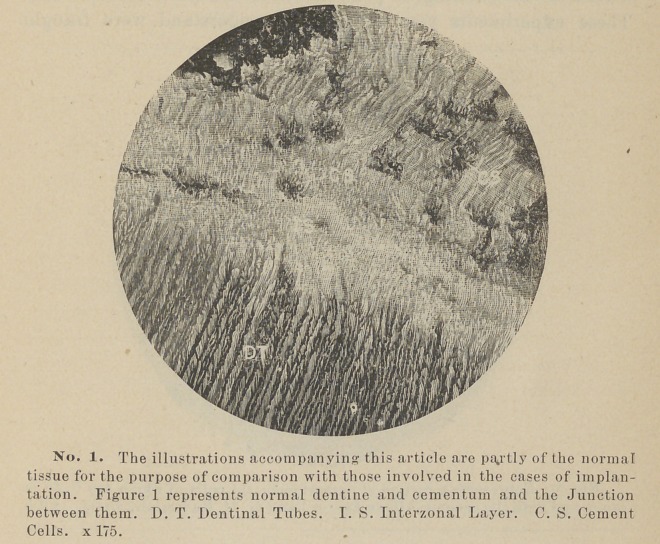


**No. 2. f2:**
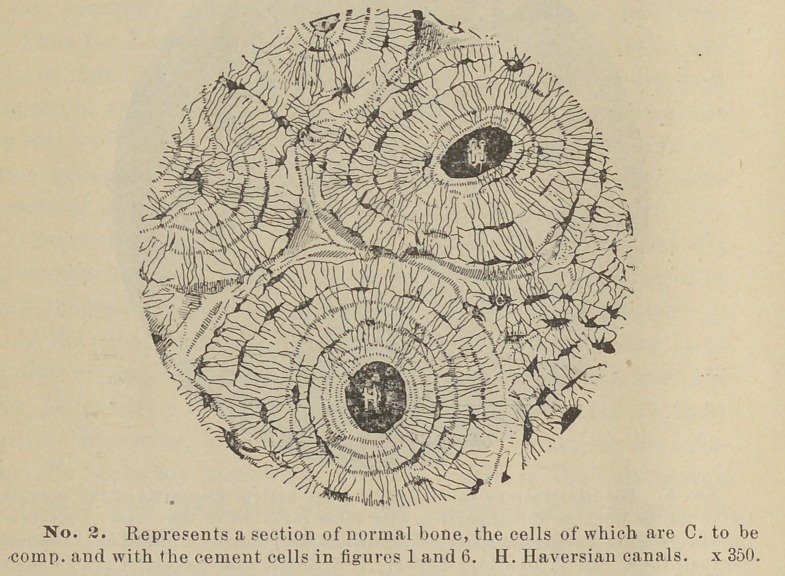


**No. 3. f3:**
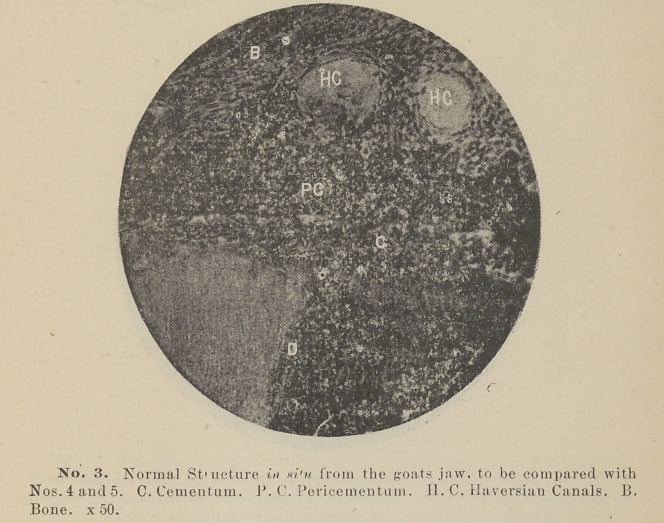


**No. 4. f4:**
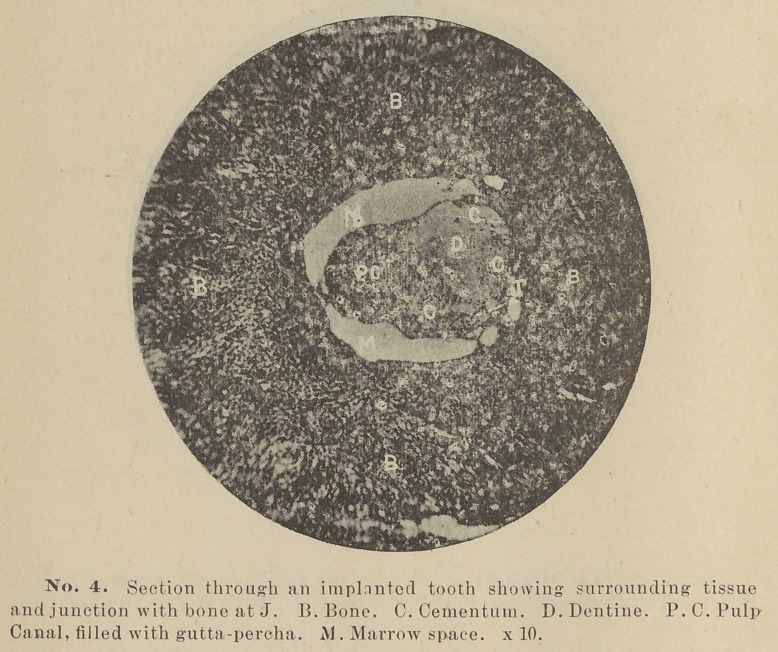


**No. 5. f5:**
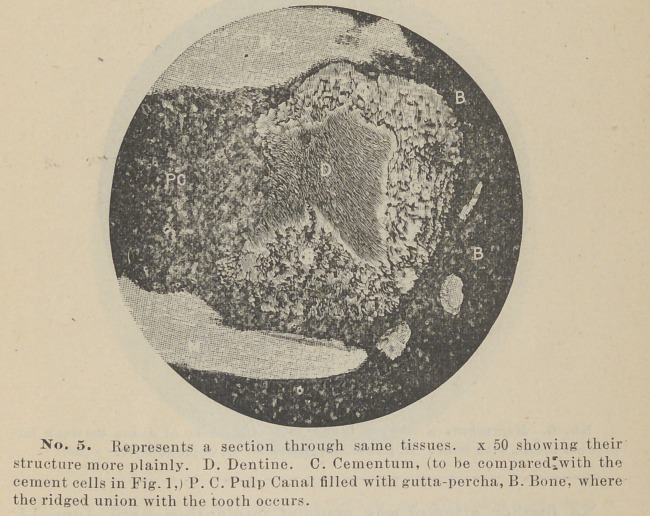


**No. 6. f6:**
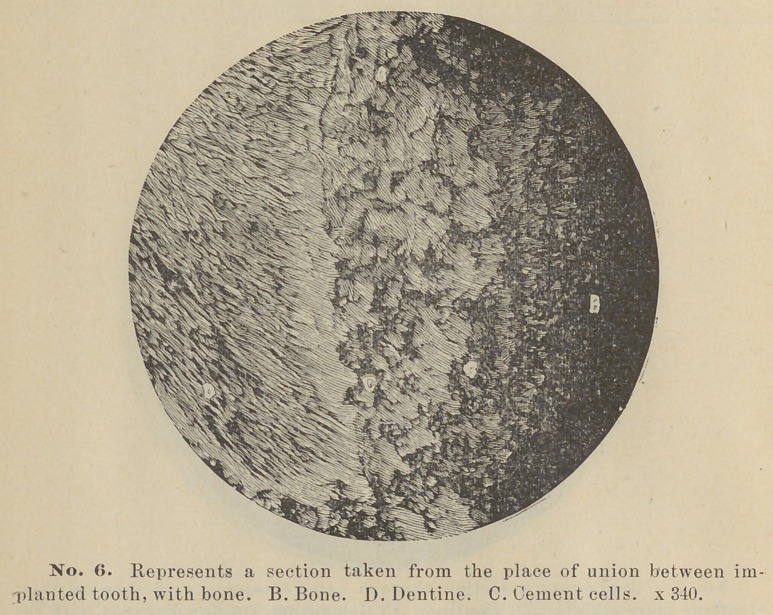


**No. 7. f7:**
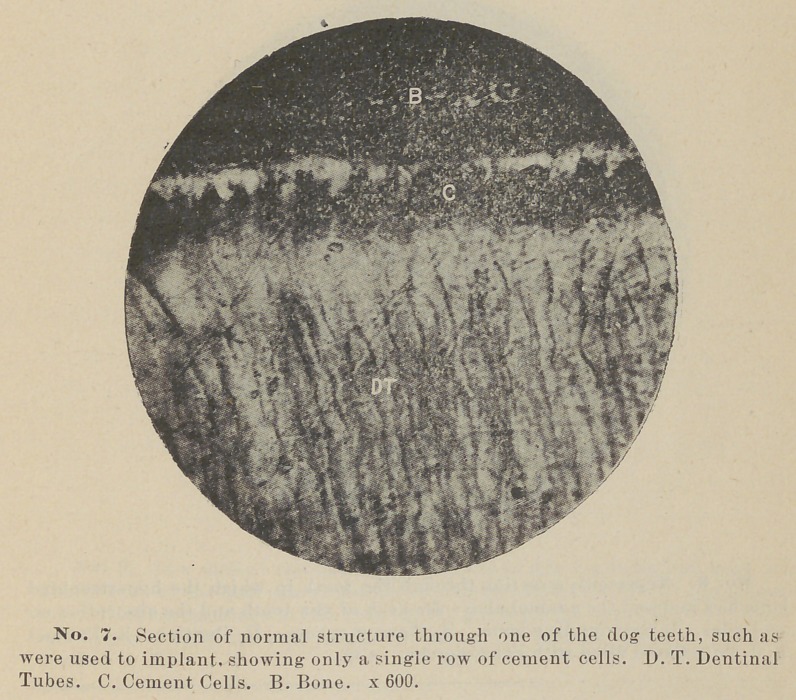


**No. 8. f8:**
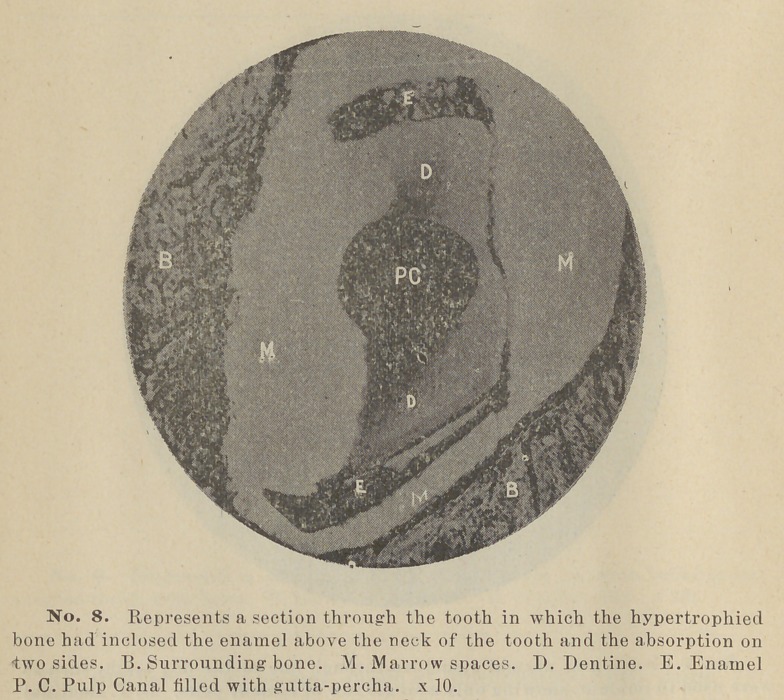


**No. 9. f9:**